# Employing an Artificial Intelligence Platform to Enhance Treatment Responses to GLP-1 Agonists by Utilizing Metabolic Variability Signatures Based on the Constrained Disorder Principle

**DOI:** 10.3390/biomedicines13112645

**Published:** 2025-10-28

**Authors:** Jakob Landau, Yariv Tiram, Yaron Ilan

**Affiliations:** Department of Medicine, Hadassah Medical Center, Faculty of Medicine, Hebrew University, Jerusalem 12000, Israel; kobi.landau@mail.huji.ac.il (J.L.); yarivtiram@hadassah.org.il (Y.T.)

**Keywords:** GLP1 agonists, obesity, stochasticity, complex systems, algorithms, constrained disorder

## Abstract

Introduction: Biological systems inherently exhibit metabolic variability that functions within optimal ranges, as described by the Constrained Disorder Principle (CDP). Deviations from these ranges, whether excessive or insufficient, are linked to adverse health outcomes. This review examines how signatures of metabolic variability can enhance GLP-1 receptor agonist therapy using artificial intelligence platforms. Methods: We conducted a comprehensive literature review examining metabolic variability across various parameters, including heart rate, blood pressure, lipid levels, glucose control, body weight, and metabolic rate. We focused on studies investigating the relationship between variability patterns and treatment responses, particularly in the context of GLP-1 receptor agonist therapy and the use of CDP-based AI systems. Results: Increased variability in metabolic parameters consistently predicts adverse outcomes, such as cardiovascular events, mortality, and disease progression. Heart rate variability shows a U-shaped association with outcomes, while blood pressure, lipid, and glucose variability demonstrate predominantly linear relationships with risk. Body weight variability is associated with cognitive decline and an increased risk of cardiovascular complications. Additionally, genetic polymorphisms and baseline metabolic profiles can influence responses to GLP-1 receptor agonists. CDP-based AI platforms have successfully enhanced therapeutic outcomes in conditions like heart failure, cancer, and multiple sclerosis by leveraging biological variability rather than suppressing it. Summary: The identification of metabolic variability signatures offers valuable predictive insights for personalizing therapy with GLP-1 receptor agonists. Artificial intelligence systems based on clinical data patterns that include these variabilities represent a significant shift toward dynamic and individualized treatment approaches. This can enhance therapeutic efficacy and help counteract drug resistance in chronic metabolic disorders, potentially improving the response to GLP-1-based therapies.

## 1. Introduction

Biological systems exhibit intrinsic variability, a principle that also applies to metabolic values [1,2]. Historically, research on metabolic variability has primarily focused on improving test accuracy by accounting for this variability, while paying little attention to the patterns and implications of the variability itself. However, recent advancements in the field have shifted the focus toward examining the adverse effects of increased variability [2,3].

Metabolic values cover a wide range of measures, including vital signs such as heart rate, blood pressure, and body mass index (BMI), as well as laboratory-based indicators like glucose levels, blood lipids (e.g., triglycerides, HDL, LDL, and total cholesterol), and uric acid levels. Metabolic variability has emerged as a significant area of interest, highlighting the importance of not only identifying target values for metabolic parameters but also evaluating the inherent variability of these parameters in patients [4].

Metabolic flexibility refers to an organism’s ability to dynamically switch between different energy substrates, such as lipids and carbohydrates, in response to changing demands and nutrient availability [5]. This flexibility is considered a key component of metabolic health, serving as an essential adaptation and regulatory mechanism. Variations in metabolic flexibility among individuals over the long term are associated with differing risks of weight gain and metabolic diseases. Differences in energy metabolism can significantly impact disease risk and the effectiveness of strategies aimed at improving metabolic health [6].

The Constrained Disorder Principle (CDP) provides a framework for leveraging biological variability to enhance the functionality of complex systems. According to the CDP, these systems operate within a dynamic range of variability, allowing them to adapt to both internal and external disturbances. Malfunctions and diseases in systems arise from either excessive variability or insufficient variability.

This paper outlines the CDP-based concept, emphasizing the necessity of maintaining a certain degree of physiological variability for optimal performance. It discusses the importance of managing this variability at lower levels. Additionally, the paper explores the potential use of an artificial intelligence (AI) system based on the CDP to optimize therapies for metabolic disorders.

Methods: We conducted a thorough literature review to examine metabolic variability across several parameters, including heart rate, blood pressure, lipid levels, glucose control, body weight, and metabolic rate. Our focus was on studies exploring the relationship between variability patterns and treatment responses, particularly in the context of GLP-1 receptor agonist therapy and the use of CDP-based AI systems.

## 2. The Constrained Disorder Principle (CDP) Offers a Framework for Leveraging Biological Variability to Enhance the Effectiveness of Chronic Therapies

Biological noise is a fundamental characteristic of all biological systems [7,8,9,10,11,12,13,14,15,16,17,18,19,20,21]. The Constrained Disorder Principle (CDP) defines complex biological systems by their inherent variability. According to this principle, all biological systems, ranging from genes to entire organs, exhibit variability that is bounded by dynamic limits. These boundaries regulate the degree of variability within a system, allowing it to adapt to both internal and external disturbances. In terms of the CDP, disease states arise from malfunctions in these boundaries, which can result in either excessive or insufficient variability within a system [20,22,23].

The CDP forms the basis for developing second-generation AI systems that leverage biological variability to address pathological conditions. By regulating the degree of variability within a biological system, it is possible to correct system malfunctions [24,25,26,27,28,29,30]. This approach can enhance functionality by dynamically adjusting the level of biological noise, increasing or decreasing it as necessary. According to the CDP, deviations from the inherent variability, whether as an increase or decrease from the baseline dynamic state, may serve as prognostic indicators for adverse health outcomes.

## 3. Increased Metabolic Variability Is Linked to a Higher Incidence of Health Issues

Metabolic variability has been studied extensively, with a specific focus on the links between increased variability and adverse health outcomes. This focus arises from observations that populations with unstable metabolic profiles experience worse clinical outcomes, including higher mortality rates [31,32,33,34], major adverse cardiovascular events (MACE) [35,36,37,38], and chronic kidney disease (CKD) [39]. Research has examined various metabolic parameters and suggested multiple pathophysiological mechanisms to explain the harmful effects of increased variability.

While most research has focused on the adverse effects of increased variability, there has been relatively little attention given to the potential risks associated with decreased variability or an excessively static profile. Few studies have extended beyond the adverse prognostic implications of heightened variability to demonstrate that reduced variability in certain metabolic factors is also associated with poorer outcomes. Although not all metabolic parameters have been clearly tied to negative results following deviations from baseline variability, this review aims to synthesize existing evidence and clarify the complex relationship between metabolic variability and health outcomes. According to the principle of CDP, variability is a built-in and necessary characteristic of living systems. Therefore, any deviation from the optimal range of variability, whether through excessive fluctuations or insufficient dynamism, may indicate pathological conditions.

## 4. Heart Rate Variability: A Subtle Equilibrium in Autonomic Regulation

Heart rate variability (HRV) reflects the function of the autonomic nervous system and is widely recognized as an essential marker of its physiological performance. Elevated HRV has been associated with poorer outcomes in specific populations. In elderly cohorts, an increase in HRV has been found to predict cardiovascular mortality, sometimes even more significantly than a decrease in variability [40]. Increased HRV was associated with higher rates of cardiovascular events and mortality [41].

Numerous studies and meta-analyses have identified a reduction in HRV as a significant negative prognostic indicator [42]. In populations of individuals who have experienced a myocardial infarction, those analyzed using Holter monitoring showed that HRV values below 50 ms, regardless of average heart rate, were the strongest predictors of mortality [43]. The observed decline in HRV in these cases was attributed to reduced vagal tone or increased sympathetic activation, both of which are closely associated with a higher risk of ventricular fibrillation events. Similarly, in stroke survivors, diminished HRV has been linked to adverse outcomes, including reduced functional recovery, higher complication rates, and increased mortality [44].

These findings support the concept of CDP and highlight the complexity of heart rate variability (HRV) as a prognostic marker, emphasizing the importance of keeping HRV within a normal physiological range. Any extreme deviation from this range, whether a decrease or an increase, has been consistently linked to adverse outcomes across diverse populations. This underscores the crucial role of balanced autonomic regulation in both health and disease.

## 5. Blood Pressure Variability and Its Effect on Prognosis

Blood pressure (BP) naturally fluctuates among individuals throughout the day, typically showing a nighttime drop. It can also vary between consecutive measurements both in the short and long term [45,46]. Numerous studies have investigated the relationship between BP and various health conditions. These studies indicate that not only do elevated BP levels pose risks, but also that high BP variability is independently linked to adverse outcomes such as increased mortality, cardiovascular events, and other complications.

Increased blood pressure variability (BPV) is linked to a higher incidence of major adverse cardiovascular events (MACE), particularly in patients with hypertension [47,48]. This association is significant even in individuals with normal blood pressure values, underscoring the clinical importance of BPV. Excessive BPV may indicate physiological dysfunctions, such as persistent sympathetic stimulation, baroreflex failure, or increased arterial stiffness [35]. The most substantial evidence suggests that long-term blood pressure variability is a predictor of poor prognosis, regardless of absolute blood pressure levels; however, short-term variability has also been linked to adverse outcomes [49]. A large meta-analysis reported a significant increase in the hazard ratio for mortality, primarily due to cardiovascular events, alongside higher occurrences of non-fatal conditions such as coronary artery disease (CAD) and cerebrovascular accidents (CVA) [49,50].

In specific subpopulations, increased BPV has emerged as a significant prognostic marker. For example, in post-CVA populations, heightened variability is strongly predictive of adverse outcomes [51]. Similarly, in patients with heart failure and preserved ejection fraction (HFpEF), BPV serves as an important prognostic indicator for cardiovascular outcomes and future hospitalizations [52]. In the chronic kidney disease population, greater BPV has been linked to accelerated renal function decline and progression to end-stage renal disease [53]. Furthermore, an extensive meta-analysis indicated that BPV may be a more potent prognostic factor than mean blood pressure values when predicting dementia and cognitive decline [54].

Conversely, there is relatively limited information regarding the potential negative implications of abnormally low BPV compared to the average blood pressure value. Short-term diurnal BPV, especially nocturnal dipping, is integral to normal physiological regulation. The lack of this expected nocturnal decline has been strongly associated with an increased risk of adverse outcomes. Consequently, individuals who do not exhibit nocturnal dipping, resulting in reduced diurnal BPV, experience unfavorable prognostic implications [46].

A recent study investigated the relationship between BPV and energy utilization in arterial smooth muscle cells [55]. The findings indicated that both excessive and insufficient BPV impaired cellular energy utilization efficiency, with an optimal range of variability being most conducive to healthy cellular function. The authors suggested that deviations from the normal range of variability may lead to cellular damage, ultimately compromising smooth muscle performance. This reinforces the notion that intrinsic variability is not a random occurrence but a fundamental characteristic of healthy biological systems. These data indicate that both excessive increases or decreases in variability, compared to the expected dynamic baseline, serve as adverse prognostic markers, according to the CDP.

## 6. Blood Lipid Variability: A Multifaceted Indicator of Cardiovascular and Metabolic Risk

Blood lipid levels are inherently dynamic and subject to variability influenced by a wide range of internal, external, and methodological factors [56]. It is crucial to differentiate between technical variability and inherent biological variability [57]. This variability varies among different populations, reflecting the complex interplay of biology, lifestyle, environment, and genetic predisposition. It is well established that even within healthy populations, there is a fundamental degree of variability in repetitive lipid levels in blood tests [58,59]. Furthermore, this variability is often greater in individuals with certain health conditions, such as diabetes mellitus [59,60].

The extent of variability also depends on the type of lipid being measured. For example, high-density lipoprotein cholesterol (HDL), low-density lipoprotein cholesterol (LDL), triglycerides (TG), and total cholesterol (TC) each exhibit distinct patterns of variability, with triglycerides generally showing higher variability compared to the others. Additionally, daily fluctuations in lipid levels have been noted, partly influenced by circadian rhythms regulated by hormonal changes and liver metabolism [59,61,62,63]. Moreover, numerous epidemiological studies have observed seasonal variations in lipid levels, with evidence suggesting that LDL and TC levels tend to be higher in winter than in summer [59]. These seasonal changes may result from factors such as temperature fluctuations, dietary adjustments throughout the year, and variations in metabolic and hormonal activity [64].

Diet and lifestyle significantly influence blood lipid variability, as dramatic changes in dietary intake [65] or extreme physical activity [66,67] can lead to abrupt shifts in lipid levels, contributing to overall variability. It is also hypothesized that genetic factors not only determine baseline blood lipid levels but may also influence their variability [68,69].

Aside from external and internal factors, testing methodology plays a critical role in variability. Elements such as fasting versus non-fasting states during sample collection [70], the biochemical methods used for analysis, variations in statistical approaches [71], and the interval between tests [72] can all impact the measured variability.

Elevated levels of TG, LDL, and TC, along with low levels of HDL, have long been associated with an increased risk of atherosclerotic cardiovascular disease and overall cardiovascular mortality [73]. This relationship has led to the development of pharmacological interventions and behavioral strategies aimed at modifying lipid profiles [74]. Historically, most investigations have focused on mean lipid values, establishing specific target concentrations for various lipid parameters under treatment regimens and lifestyle modification interventions aimed at reducing cardiovascular risk.

Recent research has shown that, in addition to absolute values, lipid variability plays a role as an independent prognostic factor. For instance, the Framingham Study indicated that variations in TC over a decade, regardless of mean values, could predict adverse cardiovascular outcomes many years later [31]. Furthermore, large-scale studies have suggested that high variability in LDL, HDL, and TG levels is a stronger predictor of risk than absolute average values [32,33,34,35,37].

In light of these findings, researchers have proposed several pathophysiological mechanisms to explain the correlation between lipid variability and poor outcomes [35]. Some studies have suggested that sudden fluctuations in lipid levels can lead to endothelial dysfunction and accelerate the development of unstable atherosclerotic plaques [35,38,69]. Others proposed that lipid variability may serve as a marker of overall physiological frailty or as an indicator of underlying systemic diseases [56,75,76].

Evidence indicates that treatments with agents like statins can reduce lipid variability. In various studies, a decrease in variability has been associated with improved prognoses, suggesting that the beneficial effects of statin therapy may be partly mediated by the responsiveness of individuals to the treatment [35]. However, higher lipid variability is still linked to increased cardiovascular risk, even among individuals who are not receiving statin therapy [69].

Variability in blood lipid levels is significant not only for cardiovascular outcomes but also for the pathogenesis of metabolic syndrome [77]. Recent research has shown that increased variability across metabolic parameters, such as TC concentrations, uric acid (UA) levels, body mass index (BMI), visceral adiposity index (VAI), and systolic blood pressure (SBP), is independently associated with a higher risk of developing Type 2 Diabetes Mellitus (T2DM) [78,79]. Another study on the elderly population found that over 6 years, significant fluctuations in TC and LDL levels were linked to increased risk of dementia and cognitive decline [75,80].

Despite interest in lipid variability, there is still limited evidence regarding the clinical significance of reducing such variability. Most studies focus on achieving stable lipid levels at the lowest possible concentrations. A study involving approximately 10,000 T2DM patients demonstrated that increased variability in TC, LDL, and HDL, but not TG, was associated with a heightened risk of mortality [32].

A detailed analysis of hazard ratios concerning the percentage variability of lipid levels reveals that TG variability behaves differently from other lipid types. Interestingly, a low variability level (below 20%, which is about normal variability) was associated with an increased hazard ratio when compared to the normal variability group. Similar findings were observed when comparing quartile 1 to quartile 2, where lower TG variability was associated with worse mortality prognoses. This phenomenon may explain why TG variability was not identified as a significant predictor after multivariate analysis; the inclusion of low variability within the normal range could obscure the expected effect when compared to high variability. This suggests that for TG, excessively low variability may also be associated with mortality [81].

According to the CDP, a certain level of lipid variability is essential for proper functioning. The data highlight the complex relationships between lipid variability and clinical outcomes, particularly the interaction between low and high variability thresholds in specific lipid categories.

## 7. Glycemic Variability Plays a Crucial Role in Diabetes Management and Has Significant Clinical Implications, Particularly Concerning Cardiovascular Risk and the Limitations of Maintaining Tight Glucose Control

Glycemic variability (GV) refers to the fluctuations in blood glucose levels over time, characterized by both the magnitude of these fluctuations (from low to high points) and their patterns (frequency and duration). It includes short-term measures, such as daily glucose, as well as long-term measures, such as HbA1c [82]. Various factors influence GV. For non-insulin-dependent patients, most of these factors relate to physiology, including postprandial hyperglycemia and the pancreas’s ability to respond to changes in blood glucose and regulate endogenous insulin levels [83]. Conversely, for patients relying on insulin, GV depends not only on physiological factors but also on adherence to insulin treatment and the method of insulin delivery [84]. For instance, self-injection using syringes often leads to less consistent glucose control compared to using insulin pumps. Among insulin pump users, those with advanced closed-loop systems can automatically adjust insulin delivery based on real-time glucose readings, resulting in improved glycemic stability and fewer fluctuations in blood sugar levels. Therefore, both patient compliance and the sophistication of insulin delivery technology are critical factors influencing glucose variability in this population [85].

Clinically, GV is a concept distinct from mean glycemia (e.g., HbA1c) and can be quantified using different statistical methods. A percentage coefficient of variation (CV) threshold of 36% is often used to distinguish between stable and unstable glycemia, with higher variability being strongly associated with an increased risk of hypoglycemia, particularly in patients treated with insulin [86,87].

Elevated GV in diabetes patients is independently associated with a higher incidence of major adverse cardiovascular events (MACE), coronary artery disease (CAD), cerebrovascular accidents (CVA), heart failure, and all-cause mortality, even after accounting for mean glycemia and other established risk factors [88]. In extensive studies, the increased risk for all-cause mortality was observed irrespective of hypoglycemia and HbA1c mean levels [89]. Mechanistic studies suggest that glycemic fluctuations contribute to cardiovascular risk by promoting oxidative stress, endothelial dysfunction, and inflammation, key factors in the development of atherosclerosis and its complications. Clinical evidence suggests that greater variability in HbA1c or fasting plasma glucose is associated with significantly higher rates of cardiovascular events and mortality compared to stable glycemic profiles [90]. Collectively, these findings support considering glycemic variability (CV > 36%) as a marker of elevated cardiovascular risk in diabetes, emphasizing the need for therapeutic strategies that stabilize glycemia and reduce fluctuations [91].

While the adverse effects of high GV are well established in the literature, there has been less focus on whether excessively reducing GV may also lead to adverse outcomes—the large ACCORD trial aimed to evaluate the effects of intensive versus standard glycemic control on cardiovascular outcomes [92]. The trial was discontinued early (after a mean follow-up of 3.6 years, rather than the planned 5.6 years) due to an observed increase in all-cause mortality in the intensive treatment group. Subsequent analyses sought to determine whether this finding was incidental or indicative of a meaningful physiological mechanism [93]. Advanced statistical evaluations ruled out hypoglycemia and rapid HbA1c reduction as primary contributors to the increased mortality. Paradoxically, the heightened risk was more significant among participants with higher mean HbA1c levels and those who showed slower reductions in HbA1c [94].

These findings support the concept of CDP and suggest that overly strict glycemic control and reduced GV may be harmful, potentially undermining physiological resilience [95]. Comparative assessments of HbA1c levels among treatment groups further indicate that a certain level of variability within the target range may be crucial for maintaining systemic homeostasis.

## 8. Weight Fluctuations and Health Risks: Reevaluating BMI as a Dynamic Metric

The Body Mass Index (BMI) is calculated by dividing a person’s weight in kilograms by their height in meters squared. It is a commonly used indicator of obesity, a complex condition influenced by both genetic and lifestyle factors. BMI variability refers to the fluctuations in an individual’s BMI measurements over time, reflecting changes in body weight relative to height. This variability can be assessed using statistical measures such as standard deviation (SD) and coefficient of variation (CV), which are derived from repeated measurements of BMI [96]. Research has shown that high BMI variability is associated with an increased risk of composite cardiovascular events, regardless of average BMI or other traditional risk factors [2]. A meta-analysis found an increased risk of myocardial infarction, cardiovascular mortality, and cerebrovascular accidents (CVA) associated with BMI variability [97].

These findings highlight the negative prognostic implications of BMI variability, consistently associating it with heightened risks of adverse cardiovascular outcomes and mortality. However, some studies suggest that BMI variability may offer potential benefits in specific subpopulations [98]. Similarly, a study conducted in Korea found a protective effect of BMI variability (measured using the adjusted standard deviation of weight, ASV) on T2DM obese individuals (baseline BMI ≥ 25). In contrast, it had a predictive value for T2DM in non-obese individuals (baseline BMI < 25) [99].

It has been suggested that the observed protective effect of BMI variability on the risk of T2DM among overweight and obese males may be due to a predominant downward trend in BMI during weight fluctuations. This suggests that the overall trajectory (slope) of BMI, rather than variability alone, is crucial in predicting the risk of T2DM [89].

The data support the CDP-based dynamic range for BMI [100,101,102]. Additional research is needed to investigate the potential benefits of BMI variability in individuals with obesity. Specifically, it is essential to examine the potential benefits of weight loss, even if it is followed by weight regain, compared to consistently maintaining a high body weight over time.

## 9. Prognostic Significance of Variability in Metabolic Rate and the Dynamics of Mitochondria

A comprehensive understanding of key metabolic parameters is crucial for accurately assessing mitochondrial variability and its physiological and pathological implications. The CDP accounts for the variability in mitochondrial function and other cellular functions [12,13,14,15,17,18,103]. Metabolic rate, typically measured in kilocalories per day (Kcal/day), refers to the total energy expenditure required by the body to sustain essential physiological processes and support physical activity. It encompasses all biochemical reactions occurring within the body. Basal metabolic rate (BMR) is defined as the amount of energy expended by the body at complete rest in a thermoneutral environment, following a 12-h fast. BMR represents the minimal energy necessary to maintain vital functions such as respiration, circulation, and cellular metabolism [104]. Resting metabolic rate (RMR) is conceptually similar to BMR but is measured under less controlled conditions. It reflects the energy expenditure of the body at rest, regardless of fasting or environmental conditions, and is typically about 10% higher than BMR [105].

Energy expenditure (EE), also measured in kilocalories, refers to the total amount of energy the body utilizes over a defined period. It includes BMR or RMR, the thermic effect of food (the energy required for digestion, absorption, and metabolism), and the energy expended during physical activity [106]. Resting energy expenditure (REE) is often used interchangeably with RMR in both clinical and research contexts, referring to the energy expended by the body at rest [107]. The respiratory exchange ratio (RER) is the ratio of carbon dioxide produced (VCO_2_) to oxygen consumed (VO_2_) during metabolism. It provides insight into substrate utilization, indicating whether carbohydrates or fats are predominantly oxidized for energy. The respiratory quotient (RQ), similar to RER, is measured at the cellular level under steady-state conditions and serves as an index of the predominant metabolic fuel source [98].

The relationship between RER and REE is crucial in clinical settings, particularly when using indirect calorimetry to measure REE. This method calculates REE by assessing VO_2_ and VCO_2_, allowing for the determination of the respiratory quotient (RQ), which is conceptually similar to RER but measured under steady-state conditions. Indirect calorimetry is generally more accurate than predictive equations, such as the Harris-Benedict equation, particularly for patients with respiratory diseases or other systemic conditions [108]. Although changes in REE do not always lead to proportional or directional changes in RER, both parameters stem from the same underlying gas exchange variables. Consequently, variability in one is likely to reflect variability in the other, even if their physiological interpretations differ [109].

Genetic factors, including both genetic and epigenetic influences, significantly contribute to the unexplained differences in RMR among individuals. The genetic heritability of RMR has been estimated to be moderate, with familial influences accounting for about 30% of the variance in RMR, independent of body composition, sex, age, thyroid function, and cardiometabolic risk factors. Additionally, epigenetic modifications, such as DNA methylation and histone acetylation, can influence gene expression related to metabolic processes, thereby contributing to interindividual variability in RMR [101,102].

Basal metabolic rate (BMR) exhibits significant variability over time within the same individual, influenced by various physiological and environmental factors. Among these, body composition plays a crucial role, with fat-free mass (FFM) identified as the most significant determinant, accounting for up to 63% of the variance in BMR. In contrast, fat mass (FM) and age contribute approximately 6% and 2% to the variance, respectively [110]. Both short- and long-term fluctuations in energy balance, such as periods of caloric deficit or surplus, can modulate BMR, reflecting the body’s adaptive metabolic responses. Previous nutritional intake and levels of physical activity further influence BMR by affecting substrate availability and energy demands [111]. Furthermore, metabolic rate variability is related to energy expenditure (EE) and the respiratory exchange ratio (RER), as well as mitochondrial activity, as indicated by the respiratory quotient (RQ) [112].

Mitochondria, the cell’s energy powerhouses, utilize oxygen to metabolize carbon intermediates from three essential nutrients: fatty acids, glucose, and amino acids. The RQ, previously mentioned, is defined as the ratio of carbon dioxide production to oxygen consumption. It ranges between 0.7 and 1.0, providing insights into mitochondrial fuel selection. A high RQ indicates a preference for glucose oxidation, while a low RQ suggests greater dependence on fat metabolism, with amino acids contributing minimally under typical conditions. In a healthy physiological state, the whole-body RQ fluctuates throughout the day, reflecting metabolic flexibility. The interaction and coordination between these competing energy sources allow mitochondria to select the most suitable fuel based on the body’s physiological needs [103,113].

The concept of metabolic inflexibility was described by studying gas exchange in the legs to compare substrate switching in healthy individuals with those who are obese or have diabetes. In lean, healthy individuals, the respiratory quotient (RQ) increases from a low level in the fasted state to a higher level when insulin levels rise. At the same time, blood glucose remains stable, mimicking the fed state. In contrast, individuals who are obese or have type 2 diabetes mellitus (T2DM) show only a slight change in RQ, continuing to oxidize a fixed mixture of fats and carbohydrates regardless of nutritional changes [114].

While variability in mitochondrial activity reflects metabolic flexibility and is generally considered a positive prognostic factor, some studies suggest that variability in the respiratory exchange ratio (RER) may indicate an unfavorable outcome [109,115]. A recent study demonstrated a positive association between body mass index (BMI), adiposity, and high variability in the RER [107,116].

The data support the CDP-based view that metabolic rate and its variability are influenced by a complex interplay of factors, including body composition, energy expenditure, hormonal influences, and genetic background. This variability is considered fundamental for normal functioning. Mitochondrial activity parameters such as the RER and RQ provide essential insights into substrate utilization and mitochondrial function. Central to these processes is RQ variability, or metabolic flexibility, the ability to efficiently shift between fat and carbohydrate oxidation, which reflects mitochondrial adaptability to varying physiological and nutritional states. According to the CDP, these measures contribute to a nuanced understanding of metabolic regulation and its variability across different conditions, providing a foundational basis for exploring metabolic health and disease [64].

## 10. GLP-1 Receptor Agonists: Connecting Therapeutic Response to Metabolic Variability Profiles

Glucagon-like peptide-1 (GLP-1) is an incretin hormone secreted by enteroendocrine cells, playing a crucial role in regulating glucose levels by stimulating insulin secretion. GLP-1 receptor agonists have become increasingly important, first in managing type 2 diabetes mellitus (T2DM) for improved blood sugar control and later as a significant breakthrough in obesity management, demonstrating remarkable efficacy for weight loss [117]. In addition to diabetes and weight loss benefits, GLP-1 receptor agonists may also have a positive impact on kidney disease, cardiovascular outcomes, non-alcoholic fatty liver disease, and blood pressure [118]. Additionally, they also enhanced heart rate variability and improved glycemic variability [119,120,121,122].

Despite their effectiveness in managing diabetes and aiding weight loss, there is significant variability in patient responses to treatment. Studies indicate that up to 20% of individuals do not respond to GLP-1 receptor agonists. Additionally, some patients may experience a partial or complete loss of the drug’s effectiveness over time, with reports of tolerance to specific effects [123,124,125].

Individual variability in responses to GLP-1 receptor agonists is affected by the primary indication for therapy. In diabetes management, prospective studies suggest that the therapeutic efficacy of these agents correlates with the capacity for endogenous insulin secretion. Patients with reduced pancreatic beta-cell function generally exhibit weaker glycemic responses, underscoring the critical role of residual beta-cell activity in mediating the effects of GLP-1 receptor activation [126].

In a study examining patients with severely uncontrolled diabetes, factors such as prior treatment history, body mass index (BMI), and average pre-prandial glucose levels within the first two days of GLP-1 therapy were found to influence efficacy [127]. Further research into hormonal biomarkers indicated that elevated baseline GLP-1 concentrations may predict a more favorable glycemic response [124].

Other findings suggest that patients with reduced endogenous GLP-1 secretion may exhibit a more pronounced therapeutic response to GLP-1 receptor agonists, as these agents can effectively compensate for the deficiency in incretin signaling. In contrast, individuals with relatively preserved endogenous GLP-1 levels may exhibit a more modest clinical improvement, possibly due to a partially intact incretin effect at baseline [128].

Genetic predisposition has also been linked to response variability. A study of an Iranian cohort identified a polymorphism (rs10305420) in the GLP-1 receptor gene that affects the degree of blood glucose reduction achieved with treatment [129]. Beyond genetic markers, microRNA (miRNA) profiles have been explored as potential indicators of treatment response. In one study, increased expression of eight miRNAs associated with T2DM correlated with a faster decline in blood glucose levels following the initiation of GLP-1 therapy [130].

GV is increasingly recognized as a significant determinant of therapeutic response to GLP-1 receptor agonists. Elevated GV may hinder the clinical efficacy of these agents, as their glucose-lowering effects are optimized in a more stable glycemic environment [131]. While GLP-1 receptor agonists have shown the potential to reduce GV, the degree of improvement varies among individuals, indicating a heterogeneous response that may be influenced by patient-specific metabolic profiles [132].

In obesity management, various demographic and physiological factors have been assessed as predictors of responses to GLP-1 receptor agonists (GLP1A). A Canadian study involving 483 patients with a body mass index (BMI) of 30 or higher used multivariate regression analysis, identifying female sex as a significant predictor of enhanced weight loss. Other variables, such as diabetic status, baseline BMI, age, and psychiatric comorbidities, did not appear to affect the therapeutic outcomes significantly [118]. A higher baseline BMI was linked to greater reductions in both BMI and body weight following treatment with GLP-1 receptor agonists [133].

Hormonal regulation has also been noted to play a role in mediating GLP-1-induced weight reduction [133]. Research indicated that higher fasting ghrelin and GLP-1 levels at baseline, along with suppressed ghrelin levels measured two hours after breakfast, were associated with more pronounced decreases in body weight [134]. Additionally, this study demonstrated that weight loss was accompanied by reductions in TG levels, with the extent of TG decline dependent on the initial hypertriglyceridemia and postprandial concentrations of glucose-dependent insulinotropic peptide (GIP) and ghrelin. A study of patients treated with GLP-1A aimed to identify predictive factors for weight loss response but found no significant predictors, including sex, race, age, ethnicity, glucose levels, level of obesity, depression, pubertal stage, and weight variability [135].

It is evident that many factors correlate with predicting response to treatment and serve as indicators of response to GLP1A therapy. Many of these variables are metabolic, including BMI, HbA1c levels, blood hormone levels, blood pressure, heart rate variability, and glucose variability [136]. Metabolic variability is a fundamental characteristic of biological systems, ensuring adaptability and resilience. However, this variability operates within an optimal range. Deviations beyond this threshold, whether excessive or insufficient, can disrupt physiological equilibrium and contribute to adverse health outcomes [136,137].

To date, most research has concentrated on the prognostic implications of metabolic variability, particularly its ability to predict outcomes. Emerging evidence has begun to explore how pharmacological interventions, particularly GLP1A, affect specific measures of variability, such as HRV and GV [138]. However, there have been no comprehensive investigations examining whether preexisting metabolic variability can serve as a predictive marker for treatment response in general, and specifically for GLP1A therapy. There is a lack of extensive research evaluating the broader effects of GLP-1A on additional metabolic parameters, which may provide insights into the drug’s mechanisms for improving clinical outcomes, including cardiovascular health [125].

## 11. Analysis of Metabolic Networks Based on Constraints and Integration of the Metabolome

According to the CDP, using constrained approaches while maintaining dynamic variability can lead to improved accuracy in modeling [23,100,101,102,139,140]. The use of the CDP-based framework is associated with improved diagnostic and treatment plans [28,30,141,142,143,144,145,146,147,148]. Constraint-based approaches have been utilized to integrate data within large-scale metabolic networks, providing insights into the metabolism of various organisms. However, these methods operate under the assumption of a steady state, making them generally unsuitable for predicting metabolite levels [149]. While constraint-based methods offer a modeling framework that aligns well with systems-based analyses, facilitating the integration of high-throughput data and primarily focusing on the stoichiometry of the included reactions, they often overlook the metabolome. The metabolome, which encompasses the levels of all measured metabolites and reaction fluxes, is a crucial indicator of the cellular metabolic state. Consequently, the steady-state assumption inherent in most constraint-based approaches limits their effectiveness in predicting metabolite levels [150].

An analysis of time-resolved transcriptomics and metabolomics data from Arabidopsis thaliana under eight different light and temperature conditions revealed that gene expression data can be effectively integrated into a genome-scale metabolic network [151]. This integration allows for the prediction of pathways known as modulators and sustainers, which are regulated based on a biochemically meaningful, data-driven null model. A follow-up analysis bridged the gap between flux-centric and metabolite-centric methods, demonstrating that under certain environmental conditions, the levels of metabolites that serve as substrates in modulators or sustainers exhibit significantly lower temporal variations compared to other measured metabolites. This finding is discussed in the context of a systems view on the plasticity and robustness of metabolite contents and pathway fluxes [152]. Furthermore, combining constraint-based modeling approaches with high-throughput data can help infer regulatory principles regarding the plasticity and robustness of metabolic behavior based solely on the stoichiometry of the underlying reactions [153].

Under specific environmental conditions, certain differential metabolic functions have substrates that tend to exhibit a lower coefficient of variation (CV) than other tested metabolites. An examination of the network topology reveals that these substrates are generally more interconnected than the other metabolites [154]. Additionally, the differential metabolic pathways typically have fewer substrates compared to the other investigated metabolic functions. The data indicate that substrate robustness can be observed under stressful environmental conditions [139]. Moreover, a method for calculating relative similarities between homologous metabolic network modules has been presented. This method, akin to classical sequence alignment, facilitates the generation of phenotypic trees that can be compared to corresponding sequence-based trees [155].

Network physiology has emerged as an essential platform for understanding physiological functions [156]. The use of CDP-based platforms can enhance the accuracy of network physiology modeling by accounting for physiological variability, thereby ensuring dynamic adaptability [23]. This approach can be applied across various domains, including gene and protein interaction networks, metabolic networks, ecological networks, and protein folding networks. Variations in the wiring patterns of metabolic networks among different organisms have been analyzed using a method that estimates similarities between these networks, similar to how biopolymer sequences are compared [157]. The relationship between network and sequence similarity provides a system-level phenotypic characteristic, network wiring, that is suitable for quantitative phylogenetic analysis. This approach can be compared to phylogenetic structures generated by molecular-level methods, such as biopolymer sequences [158].

## 12. Utilizing Frameworks Based on the Clinical Development Plan (CDP) to Enhance the Effectiveness of GLP-1 Agonists

The CDP represents a significant shift in our understanding of biological systems and therapeutic interventions. This principle suggests that biological systems operate best within a state of controlled variability, where fluctuations occur within defined limits rather than in complete randomness or strict order. In the context of metabolic health and diabetes management, this concept suggests that incorporating controlled variability into treatment regimens may enhance therapeutic outcomes compared to fixed, monotonous dosing strategies.

GLP-1 receptor agonists have become key therapies for managing type 2 diabetes, providing benefits that go beyond just controlling blood sugar levels. However, despite their proven effectiveness, many patients still experience suboptimal responses or develop resistance to treatment over time. Traditional methods focus on optimizing dosages and ensuring patient adherence, but the CDP-based approach may offer a new strategy for enhancing treatment effectiveness by incorporating controlled metabolic variability.

Previous data support the CDP-based concept that variability in therapeutic regimens can improve the response to chronic therapies and overcome the loss of responsiveness to chronic medications [57,103,142,147]. Implementing a CDP-based platform to utilize signatures of biological variability was proposed to address the partial or complete lack of response to chronic medications in various areas [57,103,142,147]. In patients with congestive heart failure who experience diuretic resistance, the use of the system has been shown to improve clinical and laboratory parameters of the disease while minimizing side effects. Additionally, it is associated with a reduction in emergency room visits and hospitalizations due to heart failure [141]. In cancer patients, the CDP-based platform enhances clinical, laboratory, and imaging measures of tumor burden while also minimizing side effects [143]. Positive effects of addressing loss of response to chronic therapies were also observed in patients with multiple sclerosis, chronic pain, anti-aging treatments, and genetic disorders [28,30,141,142,143,144,145,146,147,148].

The rationale behind the possibility of applying the CDP-based platform to GLP-1 agonist therapy is based on the understanding that metabolic systems naturally exhibit rhythmic patterns and adaptive responses. The incretin system, which includes GLP-1, operates in sync with circadian rhythms and responds dynamically to factors such as nutrition, physical activity, and other metabolic signals [159]. Rigid dosing regimens may inadvertently suppress this natural variability, potentially leading to receptor desensitization, metabolic adaptation, and reduced treatment responses over time.

A platform based on the CDP for GLP-1 agonist administration may enable controlled variations in dosing timing, frequency, and potentially dose magnitude within predetermined safe boundaries. This method simulates the natural pulsatile patterns of hormone secretion while ensuring therapeutic efficacy. For example, instead of administering a fixed weekly dose of semaglutide, the platform could vary the timing of administration within a specified window, occasionally adjust the dose within therapeutic limits, or temporarily change the injection site to prevent local tissue adaptation.

Research supports the metabolic benefits of such variability-based approaches, indicating that controlled fluctuations in metabolic parameters can enhance insulin sensitivity, improve glucose metabolism, and prevent metabolic adaptation [137,160]. Studies have shown that techniques like intermittent fasting, time-restricted eating, and varied exercise routines, all forms of controlled metabolic variability, can lead to improvements in metabolic health markers and enhance the effectiveness of pharmacological interventions [161].

The implementation of a CDP-based GLP-1 agonist platform could leverage digital health technologies, including smartphone applications and wearable devices, to monitor metabolic parameters in real-time and adjust treatment delivery accordingly. Machine learning algorithms could analyze individual response patterns and optimize variability parameters for each patient, creating a personalized approach that maximizes therapeutic benefits while ensuring safety.

Research indicates that patients with moderate, controlled glycemic variability often achieve better long-term metabolic outcomes compared to those with strictly rigid glucose control [162]. This observation aligns with the CDP concept, which suggests that optimal biological function requires controlled disorder rather than perfect stability. Additionally, the platform could incorporate lifestyle variability recommendations, including diverse meal timing, exercise patterns, and sleep schedules, all coordinated with the variable drug delivery system [163]. This holistic approach recognizes the interconnectedness of metabolic regulation and uses multiple sources of controlled variability to enhance overall treatment effectiveness.

Potential mechanisms that underlie the enhanced efficacy of CDP-based GLP-1 therapy include the prevention of receptor downregulation through varied stimulation patterns, maintenance of metabolic flexibility through controlled challenges to homeostatic systems, and enhancement of cellular adaptation capacity through intermittent stress-recovery cycles. Collectively, these mechanisms contribute to sustained therapeutic responses and potentially reduce the risk of treatment resistance.

Safety considerations for implementing such a platform necessitate careful monitoring and the establishment of appropriate boundaries for variability. The system must have robust safety protocols to ensure that variations remain within therapeutic ranges and do not compromise patient safety. Regular monitoring of key metabolic parameters, adverse events, and treatment satisfaction would be essential components of this approach.

**Figure 1:**
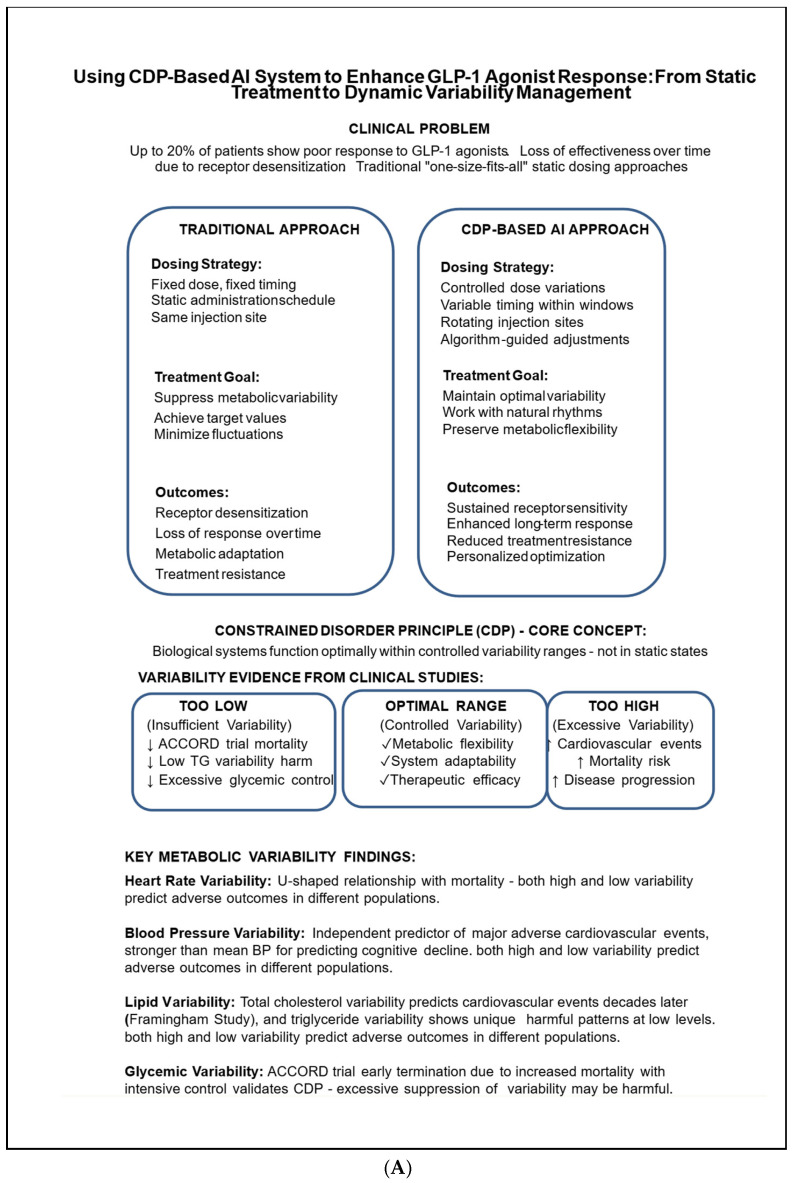

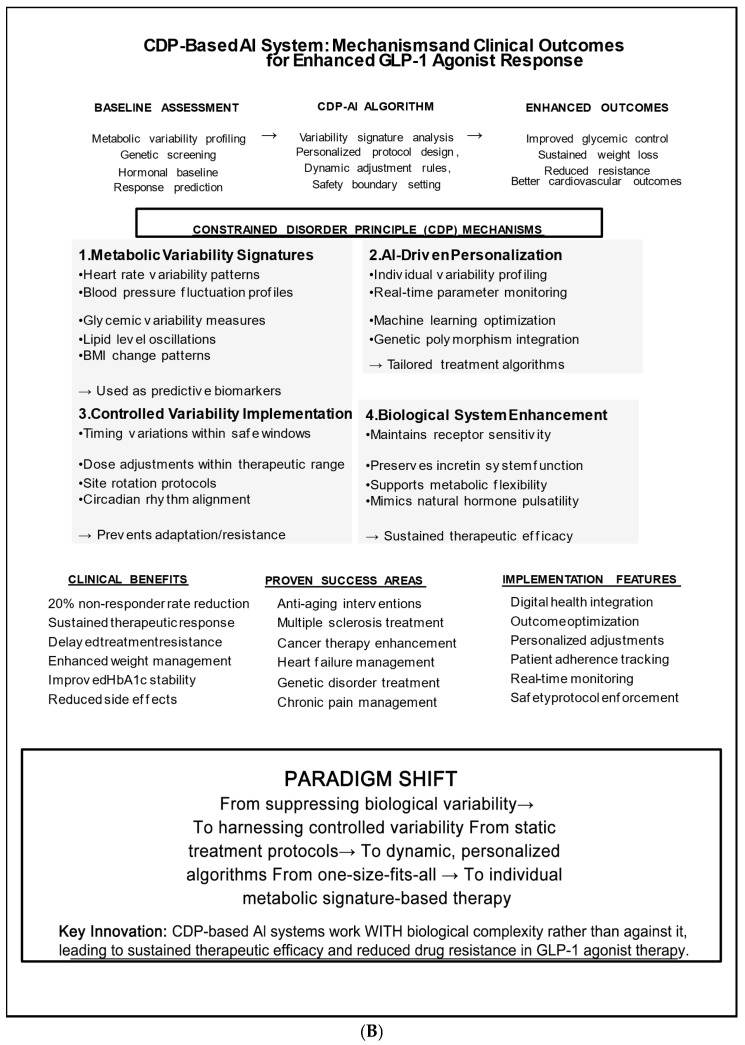
(**A**): An overview of the current approach and the concept of using CDP to enhance the effectiveness of GLP-1 agonists. (**B**): The mechanisms of the CDP-based GLP-1 approach. ↑ increase; ↓ decrease.

Figure 1A provides an overview of the current approach and the concept of using CDP to enhance the effectiveness of GLP-1 agonists. Figure 1B outlines the mechanisms by which the CDP-based system enhances drug efficacy and highlights the key concepts of the CDP-based GLP-1 approach.

## 13. Summary

This review explores the potential application of the Constrained Disorder Principle (CDP) to enhance GLP-1 receptor agonist therapy via artificial intelligence platforms that leverage metabolic variability signatures.

The research challenges the traditional medical paradigm, which focuses on minimizing biological variability, proposing instead that optimal health requires variability to be maintained within specific dynamic ranges. The CDP framework posits that biological systems function optimally when variability is kept within constrained boundaries. Disease states arise when these systems exhibit either excessive or insufficient variability, straying from their inherent dynamic baseline. This principle fundamentally reframes our understanding of metabolic health, shifting the focus from static target values to dynamic variability patterns that reflect system adaptability and resilience.

Extensive clinical evidence demonstrates that metabolic variability across multiple parameters is a powerful predictor of health outcomes. For example, heart rate variability shows complex U-shaped relationships with cardiovascular mortality, where both excessive and insufficient variability can predict adverse outcomes in different populations. Blood pressure variability independently predicts major adverse cardiovascular events, with long-term variability being particularly significant, regardless of absolute pressure levels. This relationship extends beyond cardiovascular health; blood pressure variability is a stronger predictor of cognitive decline than mean values.

Research on lipid variability reveals similarly complex patterns. While increased variability in total cholesterol, LDL, and HDL consistently predicts adverse cardiovascular outcomes and mortality, triglyceride variability exhibits unique characteristics where excessively low variability may also be harmful. Recent studies from the ACCORD trial confirm these relationships, showing that participants in the highest quartiles of lipid variability face a significantly increased risk of heart failure. Real-world evidence suggests that treatment adherence affects not only lipid levels but also their variability, with improved adherence to therapies such as PCSK9 inhibitors leading to enhanced lipid stability. Glycemic variability presents particularly compelling evidence in support of the CDP framework. Elevated glucose variability increases cardiovascular risk and mortality in patients with diabetes. In contrast, the ACCORD trial’s early termination, due to increased mortality in the intensive glycemic control group, suggests that excessive suppression of variability may also be harmful. Recent research has identified specific determinants of HbA1c variability, including ethnicity, insulin use, and triglyceride levels, with variability independently predicting mortality across different types of diabetes. Body weight variability has significant health implications that extend beyond cardiovascular outcomes. Studies indicate a strong association between weight variability and cognitive decline in older adults; those experiencing the most significant fluctuations in weight tend to show accelerated cognitive deterioration. In populations with diabetes, weight variability is a predictor of cardiovascular events and the progression of kidney disease. Additionally, research on early development suggests that fluctuations in weight during childhood can have lasting effects on the regulation of stress hormones.

The application of these findings to GLP-1 receptor agonist therapy marks a significant advancement in personalized medicine. Although these agents are effective for many patients, up to 20% do not respond adequately, leading to considerable variability in treatment outcomes. Genetic polymorphisms, particularly in the GLP1R gene, contribute to these varying therapeutic responses. Furthermore, baseline metabolic variability patterns can serve as predictive markers. Recent advanced research utilizing machine learning to evaluate satiation variability shows that genetic risk scores can help predict how individuals respond to various obesity treatments.

The integration of AI systems based on the CDP may present a novel therapeutic approach that embraces, rather than suppresses, biological variability. These second-generation AI platforms have demonstrated success across various medical conditions, including heart failure, cancer, multiple sclerosis, and genetic disorders. They achieve this by implementing dynamic treatment regimens that align with natural biological rhythms.

This body of research supports a fundamental shift in perspective toward embracing biological complexity through variability-informed treatment strategies. Future efforts should concentrate on developing comprehensive metabolic variability profiles. These profiles could guide personalized treatment selection, optimize dosing regimens, and anticipate therapeutic responses. This shift represents a transformative advancement in chronic disease management, moving away from static, one-size-fits-all treatments toward dynamic, tailored interventions that harmonize with each patient’s unique biological variability. Overall, the evidence strongly supports the possibility of implementation of CDP-based AI platforms that incorporate metabolic variability signatures to enhance GLP-1 receptor agonist therapy, overcome drug resistance, and achieve superior long-term therapeutic outcomes through personalized, variability-informed treatment approaches.

## Data Availability

No new data were created or analyzed in this study. Data sharing is not applicable to this article.

## Abbreviations

CDP	constrained disorder principle
AI	artificial intelligence
METv	variability in metabolic parameters
